# Joubert Syndrome: A Rare Radiological Case

**DOI:** 10.7759/cureus.6410

**Published:** 2019-12-18

**Authors:** Ali Akhtar, Syed Adeel Hassan, Noor Ul Falah, Maham Khan, Fahad N Sheikh

**Affiliations:** 1 Internal Medicine, Shaukat Khanum Memorial Cancer Hospital and Research Centre, Lahore, PAK; 2 Internal Medicine, Dow University of Health Sciences, Karachi, PAK; 3 Pathology, Shaukat Khanum Memorial Cancer Hospital and Research Centre, Lahore, PAK; 4 Radiology, Armed Forces Institute of Radiology and Imaging, Islamabad, PAK; 5 Internal Medicine, Sahiwal Medical College, Sahiwal, PAK

**Keywords:** delayed milestones, infantile hypotonia, joubert syndrome, molar tooth appearance, batwing configuration, oculomotor dysfunction

## Abstract

Joubert syndrome is a rare autosomal recessive neurodevelopmental disease characterized by abnormal breathing patterns composed of episodic tachypnea/apnea, hypotonia, ataxia, developmental delay, intellectual impairment, ocular impairment, renal cysts, and hepatic fibrosis. We report the case of a 4-year-old boy who presented with global developmental delay, bilateral nystagmus, and gaze instability with difficulty walking and maintaining an upright posture. A detailed examination revealed facial dysmorphic features with a depressed nasal bridge and deepened orbital sockets. Neurological examination yielded positive results for hypotonia, gait ataxia, bilateral horizontal pendular nystagmus, and a grade 1 ptosis more prominent in the right eye. However, no abnormal breathing patterns were observed in our case. Magnetic resonance imaging revealed the characteristic molar tooth sign and a batwing appearance of the fourth ventricle.

## Introduction

Joubert syndrome (JS) is an autosomal recessive neurological disorder named after Marie Joubert in 1969 [1]. It presents with abnormal oculomotor findings, hypotonia, ataxia, respiratory dysregulation, and developmental retardation owing to abnormalities of the cerebellum and brainstem [2-4]. Classic JS is characterized by the triad of hypotonia, developmental delays, and pathognomic brainstem and cerebellar malformation called the molar tooth sign (MTS) [1,5]. However, more recently, the term Joubert syndrome-related disorders (JSRD) has been adopted to describe previously distinct pathological entities with the neuroradiological feature of MTS while involving other organ systems. Based on organ involvement, JSRD is classified into six phenotypes [1,6]. Such entities include JS with renal defect, JS with ocular defect (pure JS), JS with oculorenal defects, JS with hepatic defects, and JS with orofaciodigital defects [1]. The average age at diagnosis is 33 months, and therefore, JS is to be considered a syndrome with varying phenotype thus making it difficult to diagnose the accurate subtype during the newborn period [4].

## Case presentation

A 4-year-old boy presented to the radiology department as a referred case from the department of pediatrics, where he was primarily admitted for cough, bilateral nystagmus, and gaze instability. His past medical history revealed his difficulty in walking and maintaining an upright posture. These symptoms emerged at six months of age and gradually worsened. Further questioning of the patient’s mother revealed poor and delayed developmental milestones. He achieved rollover at the age of five months and social smile at the age of three months. Currently, he is barely able to stand on his own and requires permanent support. He was delivered via an uncomplicated cesarean delivery and weighed 2.6 kg at birth. The postnatal history was negative for prolonged admission at the neonatal intensive care unit. Apart from a mild cough, there was no history of asthma, feeding difficulty, or respiratory problems. Detailed family history was taken, revealing a consanguineous marriage with a single male child. No other prior members in their family were affected.

On physical examination, the child appeared extremely thin and fragile. Mild facial dysmorphia was noted with a depressed nasal bridge and deepened orbital sockets. He weighed less than the 25th percentile (6.0 kg) on the pediatric growth chart for his age. He appeared to be awake and alert. However, when instructed, he was unable to focus his gaze on specific objects. Ocular examination revealed bilateral horizontal pendular nystagmus without myopia. Grade 1 ptosis that was more prominent in the right eye than the left was noted. The cardiovascular examination proved to be normal. A pulmonary examination revealed a prominent expiratory wheeze without chest indrawing. Examination findings of the cranial nerves, apart from the oculomotor nerve, were normal. The motor exam revealed hypotonia and ataxia with normal tendon reflexes.

**Figure 1:**
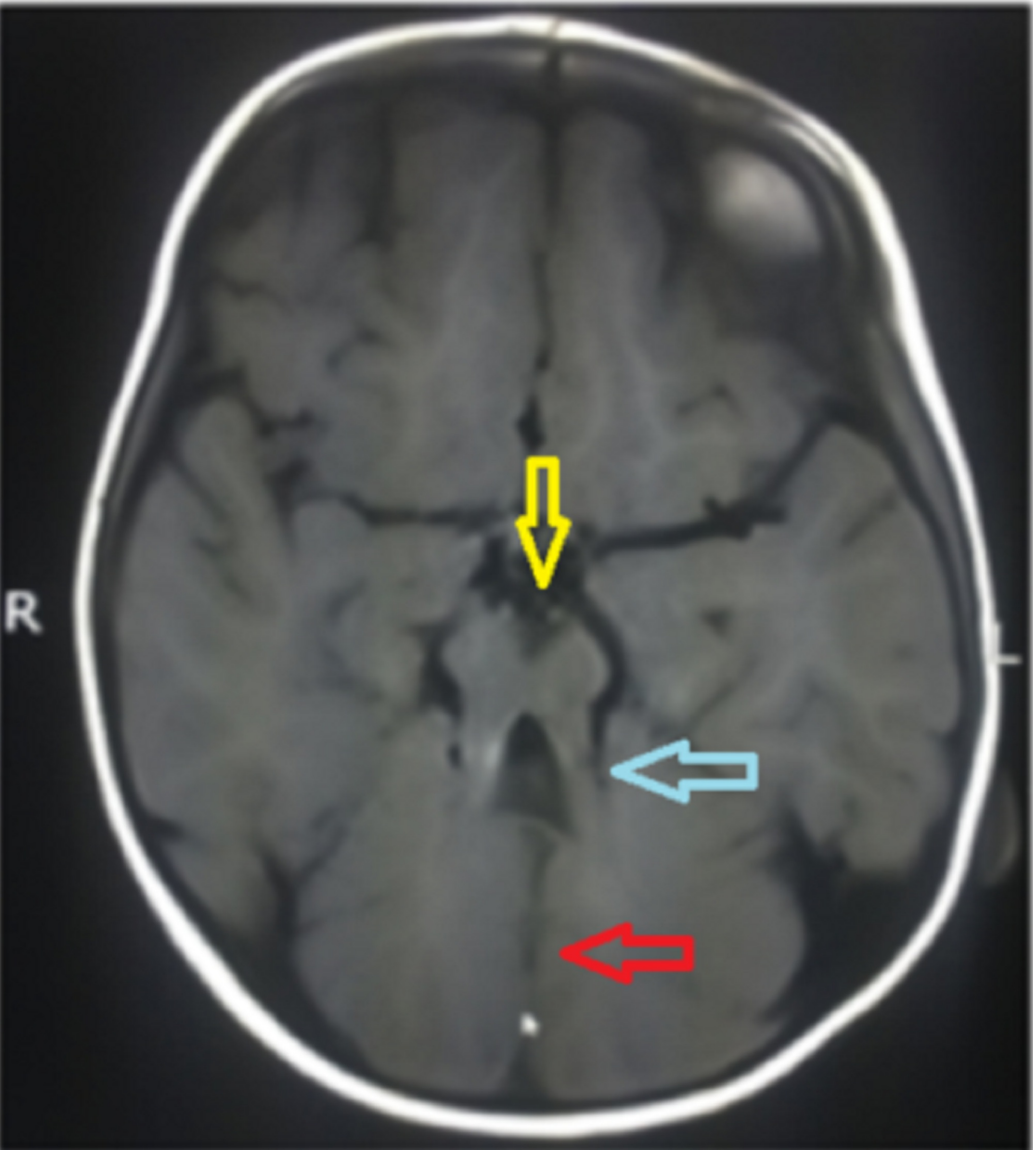
Axial T1-weighted MRI R: Right side orientation; L: Left side orientation; T1: Longitudinal relaxation time; MRI: Magnetic resonance imaging. Imaging shows deep interpeduncular fossa (yellow arrow) with thick and elongated superior cerebellar peduncles (blue arrow) imparting molar tooth appearance and aplasia of the cerebellar vermis with cerebellar hemispheres (red arrow).

**Figure 2:**
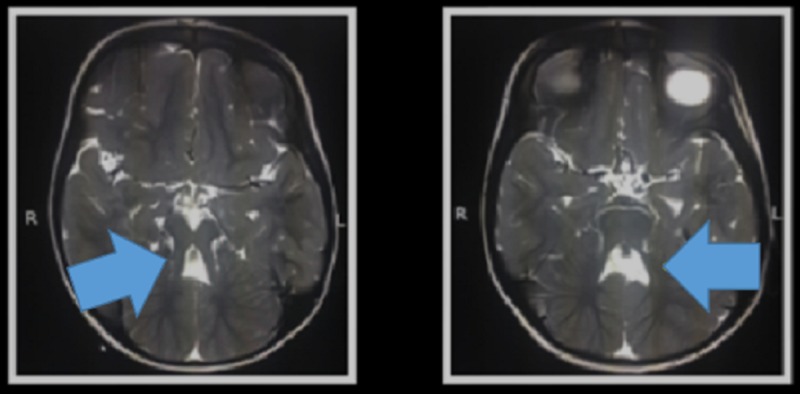
Batwing configuration of the fourth ventricle (blue arrows) R: Right side orientation; L: Left side orientation.

**Figure 3:**
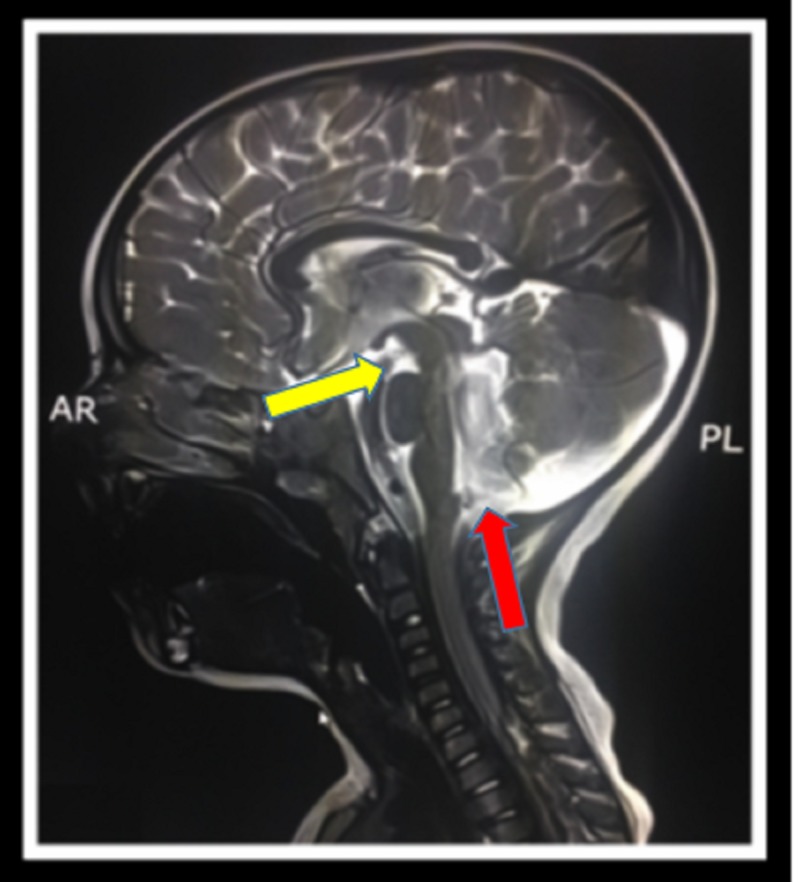
Sagittal T2-weighted MRI AR: Anterior; PL: Posterior; T2: Transverse relaxation time; MRI: Magnetic resonance imaging. Imaging depicts a deep interpeduncular fossa (yellow arrow) and prominent fourth ventricle (red arrow).

A full series of magnetic resonance imaging (MRI) scans were conducted at our radiology division to determine the cause of the delayed milestones and to figure out the source of the hypotonia and bilateral nystagmus. The axial T2-weighted MRI revealed total aplasia of the cerebellar vermis with prominent, thickened, and elongated superior cerebellar peduncles forming a characteristic molar tooth appearance. Furthermore, the fourth ventricle appeared enlarged and triangular, giving it a slight batwing appearance (Figures 1-3). Based on these clinical findings, MRI scans, and family history, a diagnosis of JS was forwarded to the clinical team.

## Discussion

JS is known to be underreported as having a prevalence of less than one in 100,000. Until 2009, approximately 200 cases of JS had been reported worldwide, with a further 12 cases being reported to date [2,3]. It was originally described in 1968 when four siblings presented with malformation of the cerebellar vermis presenting with intellectual disability, ataxia, abnormal eye movements, and episodic hyperpnoea [1].

Cilia are either motile or non-motile organelles that, via ciliogenesis, transport proteins in either direction. A defect in ciliogenesis results in the abruption of various signaling pathways such as Wnt, sonic hedgehog, planar cell polarity, and directional movement. One such major transduction pathway plays a vital role in regulating neuronal cell proliferation and axonal migration [1]. JS is known to have a genetic origin with an autosomal recessive pattern of inheritance. Of the 34 genes known to cause JS, 33 are autosomal recessive, and one is X-linked [5]. A defect in genes encoding for cilium proteins results in the clinicopathological manifestations of JS and JSRD [1,2]. Clinically, it is characterized by intellectual impairment, hypotonia, ataxia, abnormal eye movements, and abnormal breathing patterns. The essential clinical features of JS are summarized infantile hypotonia, developmental delay, and one or both of the following: abnormal eye moment and/or respiratory dysregulation [4,5].

The term JS is reserved for individuals who fulfill only the diagnostic criteria of developmental delay, abnormal ocular movements, radiological evidence of molar tooth sign, and cerebellar vermis changes [6]. The term JSRD refers to conditions that share the clinical features and radiological evidence of MTS and further involve other organs outside the central nervous system [6,7]. JSRD are now classified according to a newly adopted classification system. The system proposed classifies JSRD into six sub-groups based on genotype-phenotype correlation [1]. These include pure JS, JS with ocular defect (JS-O), JS with renal defect (JS-R), JS with oculorenal defects (JS-OR), JS with hepatic defect (JS-H), and JS with orofaciodigital defects (JS-OFD) [1,4]. These six subtypes and their associated clinical features are described in Table 1 [1].

**Table 1:** Joubert syndrome clinical features by subtype JS: Joubert syndrome; AHI 1: Abelson helper integration site 1; NPHP 1: Nephrocystin 1; TMEM 67: Transmembrane protein 67; CEP 290: Centrosomal protein 290; RPGRIP1L gene: RPGRIP 1 like protein gene.

Clinical Subtype	Clinical Features
Pure JS	Hypotonia, ataxia, developmental delay, molar tooth sign
	No retinal or liver involvement
	No major gene associated with the phenotype
JS with ocular defect	Molar tooth sign, neurological features
	Retinal dystrophy and Leber’s congenital amaurosis
	AHI1 gene most commonly mutated (20% of cases)
JS with renal defect	Molar tooth sign
	Nephronophthisis
	Absence of retinal involvement
	NPHP1 and RPGRIP1L genes commonly mutated
JS with hepatic defect	JS features
	Congenital hepatic fibrosis, chorioretinal or optic nerve colobomas, and nephronophthisis
	TMEM67 gene mutated in 70% of cases
JS with oculorenal defects	Neurological signs
	Retinal dystrophy, nephronophthisis
	CEP290 gene mutated in 50% of cases
JS with oro-facio-digital defects	Neurological features of JS
	Lobulated tongue, multiple oral frenula, mesoaxial polydactyly with y-shaped metacarpals, cleft lip/palate
	Hypothalamic hamartoma or congenital absence of the pituitary gland

The examination reveals facial dysmorphic features, including a large head, prominent forehead, rounded eyebrows, epicanthal folds, ptosis, upturned nose with evident nostrils, and low-set tilted ears [8]. Hypotonia and intellectual disability are deemed to be consistent features of JS. The breathing pattern in such patients includes hyperventilation worsened by stimulation, followed by periods of apnea or episodic hyperpnea alone [6]. Respiratory dysregulation is known to be more prominent during the neonatal period and diminishes by six months of age [3,7]. However, irregular breathing is not considered a consistent feature; it was reported in only 68% of cases by Pellegrino et al., and 44% of cases by Kendall et al. [9,10]. Abnormal eye movements manifest due to underlying oculomotor dysfunction [8]. Oculomotor apraxia is the most characteristic and frequent eye finding. It presents as the inability to track smooth pursuits and failure of the vestibulo-ocular reflex [1]. The retina is also commonly involved resulting in fundus flavus, congenital retinal dystrophy, chorioretinal coloboma, and perimacular and retinal blindness [1,8]. Other associated eye findings include nystagmus, strabismus, and ptosis. Renal involvement tends to occur in 25% of patients. Nephronophthisis is the most common form of renal involvement resulting in thickening of the basal membrane of the tubular epithelium, interstitial fibrosis, and small cysts at the corticomedullary junction [1]. These patients tend to present with signs of polydipsia and polyuria until chronic renal insufficiency manifests in the second decade of life. Hepatic involvement originates from malformation of the ductal plate and fibrous enlargement of the portal tracts. These subtypes of patients tend to present with hepatosplenomegaly, portal hypertension, cirrhosis, esophageal varices, and elevated liver function tests [1].

From a radiological perspective, a characteristic molar tooth sign can help guide the diagnosis of JS [6]. MTS results from midline hypoplasia of the cerebellar vermis, incomplete fusion of the halves of the vermis, abnormally deep interpeduncular fossa, and thick superior cerebellar peduncles [1,2,5-7]. The batwing or umbrella sign can also be seen due to the hypogenesis of the cerebellar vermis resulting in dilatation of the fourth ventricle [2,6,7]. Histopathological studies have confirmed that the gross appearance of the brainstem and cerebellum results due to the fragmentation of the dentate nucleus [1,4]. The pontomesencephalic junction is dysplastic with abnormal decussation of the superior cerebellar peduncle. There is also a marked decrease in the neurons of the basis pontis and reticular formation [4]. Another diagnostic feature noted on imaging is known as a buttock sign; it is formed due to the absence of the posterior verman lobe, leaving the cerebellar hemispheres separated by a cleft. Other findings include corpus callosum dysgenesis and moderate lateral ventricular enlargement [2]. A detailed clinical history comprising the classical triad of JS and characteristic MTS sign on MRI are sufficient to confirm or exclude the disease. Upon diagnosis of JS/JSRD, the child should be investigated for possible multiorgan involvement. Ocular investigations include evaluation of visual acuity, ocular motility, slit lamp examination, fundus oculi, and electroretinogram [1]. Standard urine analysis with an emphasis on urine specific gravity should be considered. An abnormal urine specific gravity warrants a challenge test to assess the urine concentrating ability. An abdominal ultrasound can rule out hepatic fibrosis and evidence of renal structural changes [1]. In pregnant females, a diagnosis of JS is made possible prenatally by serial ultrasound imaging starting at 11 to 12 weeks gestation. This should be followed by an evaluation of cerebellar and fetal anatomy through 20 weeks of gestation and fetal MRI imaging at 20 to 22 weeks’ gestation [6].

Management strategies are aimed at supportive and symptomatic treatment. Special care needs to be taken to manage respiratory and feeding problems. Cognitive difficulties require appropriate rehabilitation strategy, and regular follow-up [1]. These patients are extremely sensitive to the respiratory depressant effects of anesthetic agents such as opiates and nitrous oxide and therefore, require apnea monitoring for intensive care management [2]. The prognosis is dependent on the type and extent of organ involvement. Developmental outcomes vary and can result in patients who die young, patients who survive with developmental delay and visual/motor deficit, and patients whose developmental quotients fall within the mildly delayed range (70 to 80) [8]. Language and motor skills are delayed in JS/JSRD patients. Such patients require special schooling to learn specific job skills and to work in a protected environment [8]. Annual screening as per diagnostic protocol is recommended for such individuals.

Our patient presented with prototypical findings of hypotonia manifested as difficulty maintaining an upright posture since the age of six months. He also presents with features of bilateral nystagmus and oculomotor dysfunction. However, no respiratory pattern dysregulation was noted in our patient; the late diagnosis of the child may account for an improvement in breathing dysregulation with age. Also consistent with the diagnosis of JS were the MRI findings of molar tooth appearance, the batwing appearance of the fourth ventricle, and hypoplasia of the cerebellar vermis. Due to the absence of multi-organ involvement, the diagnosis of JSRD was omitted, and that of pure JS was made. A possible role of consanguinity was also reported by İncecik et al. at a rate of 63.6% [7]. Consistent with İncecik et al., our patient also had a positive history of parental consanguinity.

## Conclusions

JS presents with the clinical features of respiratory dysregulation, infantile hypotonia, developmental delay, nystagmus, oculomotor disturbance, and intellectual impairment. Due to the variability in clinical phenotypes, a delayed diagnosis is possible. Diagnosis requires essential diagnostic criteria in clinical history, MRI findings, and a multi-gene panel. The prototypical MRI finding is a molar tooth appearance with concomitant cerebellar vermis hypoplasia and a batwing configuration of the fourth ventricle. Management of such cases includes easing respiratory and feeding difficulties, along with rehabilitation for cognitive and behavioral difficulties.
